# Complete Mitogenome of a Leaf-Mining Buprestid Beetle, *Trachys auricollis*, and Its Phylogenetic Implications

**DOI:** 10.3390/genes10120992

**Published:** 2019-12-01

**Authors:** Lifang Xiao, Shengdi Zhang, Chengpeng Long, Qingyun Guo, Jiasheng Xu, Xiaohua Dai, Jianguo Wang

**Affiliations:** 1 Leafminer Group, School of Life Sciences, Gannan Normal University, Ganzhou 341000, China; xiaolifang@cnu.edu.cn (L.X.); 18645133878@163.com (S.Z.); lchp617650632@gmail.com (C.L.); guoqingyun@gnnu.edu.cn (Q.G.); 0900048@gnnu.edu.cn (J.X.); 2 National Navel-Orange Engineering Research Center, Ganzhou 341000, China; 3 College of Agriculture, Jiangxi Agricultural University, Nanchang 330045, China; jgwang@jxau.edu.cn

**Keywords:** Buprestoidea, Byrrhoidea, Elateroidea, Scirtoidea, Elateriformia, mitochondrial genome, phylogeny

## Abstract

A complete mitogenome of *Trachys auricollis* is reported, and a mitogenome-based phylogenetic tree of Elateriformia with all protein-coding genes (PCGs), rRNAs, and tRNAs is presented for the first time. The complete mitochondrial genome of *T. auricollis* is 16,429 bp in size and contains 13 PCGs, two rRNA genes, 22 tRNA genes, and an A + T-rich region. The A + T content of the entire genome is approximately 71.1%, and the AT skew and GC skew are 0.10 and −0.20, respectively. According to the the nonsynonymous substitution rate to synonymous substitution rates (Ka/Ks) of all PCGs, the highest and lowest evolutionary rates were observed for atp8 and cox1, respectively, which is a common finding among animals. The start codons of all PCGs are the typical ATN. Ten PCGs have complete stop codons, but three have incomplete stop codons with T or TA. As calculated based on the relative synonymous codon usage (RSCU) values, UUA(L) is the codon with the highest frequency. Except for trnS1, all 22 tRNA genes exhibit typical cloverleaf structures. The A + T-rich region of *T. auricollis* is located between rrnS and the trnI-trnG-trnM gene cluster, with six 72-bp tandem repeats. Both maximum likelihood (ML) and Bayesian (BI) trees suggest that Buprestoidea is close to Byrrhoidea and that Buprestoidea and Byrrhoidea are sister groups of Elateroidea, but the position of Psephenidae is undetermined. The inclusion of tRNAs might help to resolve the phylogeny of Coleoptera.

## 1. Introduction

The Buprestoidea superfamily comprises two families: Buprestidae and Schizopodidae. Schizopodidae is a small family with only seven species in three genera [1], whereas Buprestidae is the eighth largest family in Coleoptera, with approximately 15,000 species in 522 genera [2,3]. Thus far, only six mitogenome sequences of Buprestoidea have been submitted to the NCBI database, with four genera of Buprestidae and no record of Schizopodidae [4]. The genus *Trachys* F. belongs to the tribe Tracheini (Elateriformia: Buprestoidea: Buprestidae: Agrilinae), with 637 species in the Afrotropical, Australasian, Oriental, and Palaearctic regions [3]. The tribe Tracheini contains mainly small and cuneiform leaf- or stem-mining beetles [5,6]. *Trachys auricollis* Saunders 1873 includes two synonym species: *T. sauteri* Kerremans 1912 and *T. freyi* Théry 1942. *T. auricollis* is widely distributed in Asia [3,7], and damage due to either larval mining or adult feeding can lead to a reduction in plant photosynthesis and growth [8]. As a specialist herbivore, the leaf-mining beetle is also the most promising biocontrol agent for kudzu [9], a seriously invasive plant in the USA [10]. There are no bioinformatic studies on the mitogenome of *T. auricollis* to date.

**Figure 1:**
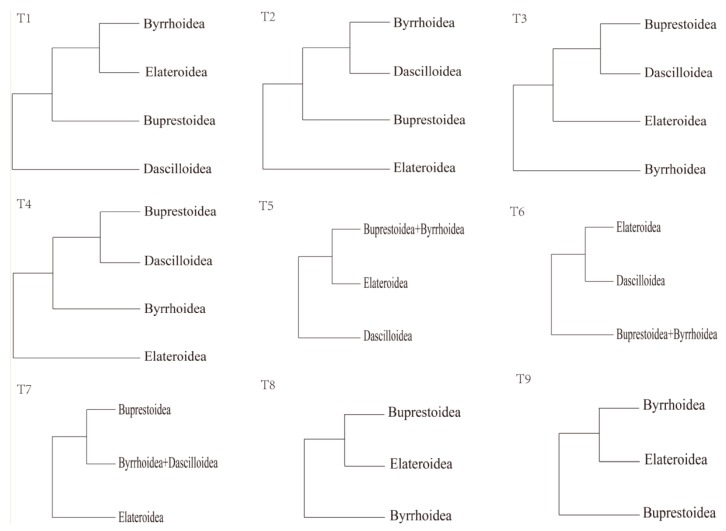
Nine gene-based topologies among four superfamilies of Elateriformia. Topologies are derived from: T1 refs. [16,17], T2 ref. [26], T3 ref. [27], T4 ref. [26], T5 refs. [28,29,30], T6 ref. [18], T7 ref. [26], T8 ref. [31], and T9 refs. [23,24,25].

With highly specialized larvae and adults, Buprestoidea is problematic about its monophyly [11]. Moreover, there are different views regarding the phylogenetic relationship of Buprestoidea with other Elateriformia superfamilies such as Byrrhoidea, Elateroidea, and Dascilloidea (Figure 1) [11,12,13]. In contrast to the traditional placement of Scirtoidea in Elateriformia [13,14,15], Scirtoidea/Scirtiformi is now treated as one basal group of Polyphaga [16,17,18]. Nosodendridae is occasionally placed in Elaterioformia [16,18]. The relationship between Buprestoidea, Byrrhoidea, and Elateroidea is the focus of our study. Some taxonomists have argued that Buprestoidea is a monophylum with a position either inside or outside byrrhoid lineages, with Elateroidea being a sister clade to Buprestoidea and Byrrhoidea (Figure 1) [12,13,14,19,20,21,22]. However, several recent studies have indicated that Byrrhoidea and Elateroidea have a close relationship and that Buprestoidea is located outside their group [17,23,24]. Conversely, according to Duan et al. (2017), Buprestoidea is the basal Polyphaga branch and is isolated from all other Elateriformia superfamilies [25].

Insect mitogenomes are closed-circular molecules of approximately 16 kb in length, with 37 genes and a noncoding A + T-rich region [32,33,34,35,36]. Mitogenomes have been widely utilized to analyze population genetics, phylogeography, and molecular phylogenetics at different taxonomic levels [37,38,39,40]. Furthermore, mitochondrial protein-coding genes (PCGs), rRNAs, tRNAs, and their combinations have been adopted to explore species differentiation and phylogenetic problems [41,42,43]. tRNAs are traditionally considered to be inappropriate phylogenetic markers because of their short length, duplication, horizontal transfer, or even change in specificity [44]. However, the overall set of tRNAs in each complete genome could reflect a stable phylogeny [44]. tRNA sequence and structure might provide additional useful information to solve phylogenetic problems, especially at higher taxonomic levels [44,45,46]. The inclusion of tRNAs has improved phylogenetic resolution in several insect groups including Diptera, Orthoptera, Neuropterida, and Lepidoptera [43,47,48,49]. Thus far, however, there has been no adoption of tRNAs to resolve the Elateriformia phylogeny (Table 1). This is the first report that uses all PCGs, rRNAs, and tRNAs to dissect phylogenetic relationships in Elateriformia, especially the complicated relationship among Buprestidae, Elateroidea, and Byrrhoidea. Our findings will contribute to further studies on the identification, phylogeny, and evolution of leaf-mining jewel beetles.

## 2. Materials and Methods

### 2.1. Sampling and DNA Extraction

Specimens of *T. auricollis* were collected on 18 August 2017, at Jiulianshan, Jiangxi Province, China (geographic coordinates: 24°34’11.99’’N, 114°26’24’’E). Adults were stored in 100% ethanol at −80 °C. Samples were cataloged in the voucher collection of the Leafminer Group, School of Life Sciences, Gannan Normal University. *T. auricollis* specimens were sent to Shanghai Personal Biotechnology Co., Ltd. for mitogenome sequencing on 22 August 2017. Total genomic DNA was extracted from the head tissue of a single specimen using the CTAB method. DNA was preserved at −20 °C and used for sequencing. The mitogenome sequence (MH638286) was submitted to the GenBank on 17 July 2018, as Submission2134492.txt.gz. 

### 2.2. Genome Sequencing and Analyses

The total mitogenome of *T. auricollis* was obtained by next-generation sequencing using the whole-genome shotgun (WGS) strategy based on the Illumina MiSeq platform. Genomic DNA libraries were prepared using the Rapid Plus DNA Lib Prep Kit for Illumina. We then acquired and checked the raw data, including library insert fragments (approximately 400 bp); paired-end reads (2 × 251 bp), approximately 16,429 bp in length were obtained. The read numbers and total bases for *T. auricollis* are 5,331,476 bp and 1,418,148,881 bp, respectively, with approximately 485 bp of missing sequence. The contigs and scaffolds of highly qualified sequences were determined using A5-miseq v20150522 [50] and SPAdesv3.9.0 [51]. Sequences with high sequencing depth were then compared with the NCBI nt library using BLASTN (BLAST v2.2.31+) [4] to select mitochondrial sequences resulting from different assemblies. MUMmer v3.1 [52] was used to perform collinear analysis, confirm the contig positions, and fill the gaps between contigs. Pilon v1.18 [53] was applied to correct the results and obtain the final mitochondrial sequences (*.fasta). The mitogenome was annotated on the MITOS web server (http://mitos.bioinf.uni-leipzig.de/index.py), and coding regions were manually verified by comparison against the NCBI database. All tRNA gene structures were predicted and determined by tRNA scan-SE or MITOS. Two rRNAs and all PCGs were annotated by alignment with homologous genes from another unpublished *Trachys* mitochondrial sequence (Table 2) using Geneious R11 [54]. Tandem repeats in the putative control region were assessed by Tandem Repeats Finder (http://tandem.bu.edu/trf/trf.html). MEGA version 7.0 [55] was employed to calculate the A + T content, the nonsynonymous substitution rate to synonymous substitution rate (Ka/Ks) ratio, and the relative synonymous codon usage (RSCU) for PCG analysis. Genome organization and base composition, PCGs, codon usage, transfer RNAs, ribosomal RNAs, A + T-rich region, intergenic spacers, and overlapping regions of the mitogenome were compared between *T. auricollis* and *T. troglodytiformis*. The document ‘linear_order.txt’ obtained from the PhyloSuite was used to check gene rearrangement through the CREx website (http://pacosy.informatik.uni-leipzig.de/crex/) [56].

### 2.3. Phylogenetic Analyses

Phylogenetic analyses were performed based on the concatenated nucleotide sequences of all 13 PCGs, both rRNAs and 22 tRNAs for Elateriformia species, with Scirtoidea species used as outgroups. All available Buprestoidea species, Byrrhoidea species, Scirtoidea species, and Elateroidea families were covered. Because of the high abundance available mitogenomes for Elateroidea, we selected one representative species for each Elateroidea family. The representatives should be annotated as VERIFIED species, with the largest mitogenome sequence length. All mitogenomes chosen were complete or nearly complete in order to obtain all 37 genes. With seven buprestoid species, nine byrrhoid species, eight elateroid species, and five scirtoid species (Table 2), the number of species in each superfamily was similar and thus were balanced for topological construction. 

The mitogenomes were obtained on 24 September 2019, from NCBI GenBank (Available online: http://www.ncbi.nlm.nih.gov). All mitogenome sequences were imported and standardized in PhyloSuite [57]. All PCGs, tRNAs, and rRNAs were extracted and aligned with MAFFT [58]. The concatenation of multiple alignments was performed, and a partition file was prepared; the partitioning scheme was obtained with PartitionFinder [59]. A greedy algorithm was adopted with the criterion of AICc to select the best-fit substitution model: GTR + G for the maximum likelihood (ML) tree and GTR + I + G for the Bayesian (BI) tree. ML tree was constructed with the IQ-Tree method [60] and BI tree with MrBayes methods [61]. Bootstrap analysis in IQ-Tree for each node was calculated using 1000 replications, with the MCMC setting in MrBayes for Generations for 2,000,000 times and a sampling frequency of 1000 replications. The phylogenetic trees were drawn using the software FigTree v1.4.3 [62].

## 3. Results and Discussion

### 3.1. Genome Organization and Base Composition

The complete mitogenome of *T. auricollis* (GenBank: MH638286) is 16,429 bp in size, with an A + T content of 71.1%. As with other beetle mitogenomes, the nucleotide composition of the *T. auricollis* mitogenome has an obvious A + T bias. In general, the A + T content of Buprestoidea is lower than that of other superfamilies (Table 2).

**Figure 2:**
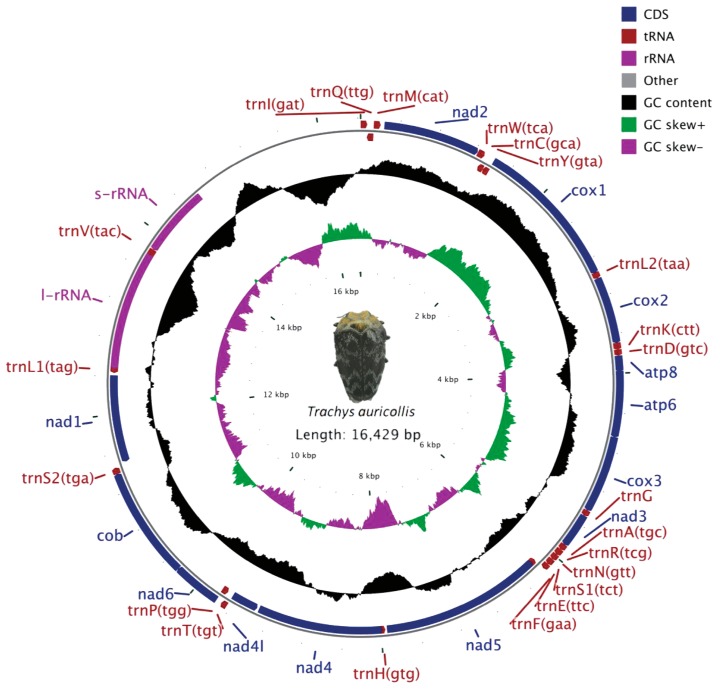
Circular map of the mitochondrial genome of *T. auricollis*. Genes outside the circle are transcribed in a clockwise direction, whereas those inside the circle are transcribed counterclockwise. Protein-coding genes (PCGs) are in blue, tRNA genes are in red, and rRNA genes are in purple. The second circle shows the GC content, and the third shows the GC skew. The GC content and GC skew are plotted as the deviation from the average value of the entire sequence.

The mitogenome consists of 37 genes (13 PCGS, 22 tRNAs, and two rRNAs) and an A + T-rich region. Twenty-three genes (9 PCGs and 14 tRNAs) are located on the major strand (N-strand) and 14 genes (4 PCGs, 8 tRNAs, and 2 rRNAs) on the minor strand (J-strand) (Figure 2 and Table 3). The gene arrangement and orientation are similar to the typical beetle mitochondrial genome [38,72].

The AT and GC skews of the complete mitogenome of *T. auricollis* were calculated, and the highest AT skew and GC skew values were found in the control region (CR) (0.04) and rrnL (−0.15). The AT skew and GC skew values of all PCGs in *T. auricollis* range from −0.35 (nad1) to 0.041 (atp8) and −0.31 (nad3) to 0.27 (nad5), respectively. Compared with all PCGs of *T. troglodytiformis*, some differences in the AT skew and GC skew values for cox1 and nad3 were observed (Table S1, Figure S1). The base composition might influence the values of AT skew and GC skew [73]. Related studies have suggested that for substitution models incorporating strand bias, mitochondrial replication might influence the GC skew in PCGs between the leading and lagging strands [74,75], and AT skew and GC skew have been determined to be a signal of transformation between the leading and lagging strands [72].

### 3.2. Protein-Coding Genes

All 13 PCGs of *T. auricollis* comprise 11,097 bp (Table 3), which can be translated into 3317 amino-acid-coding codons, excluding stop codons (33 bp). The A + T content of all PCGs in the *T. auricollis* genome is 69.4%, ranging from 63.9% (cox1) to 77.4% (atp8). Compared with *T. troglodytiformis* (11,134 bp), A + T bases account for approximately 73% of all PCGs, ranging from 68.8% (cox1) to 80.4% (nad6) (Table S1, Figure S1). However, compared to most other beetle groups [25,38], low A + T contents are found in jewel beetles (Table 2).

All PCGs of *T. auricollis* initiate with the typical mitogenome ATN codon (Table 3); conversely, for *T. troglodytiformis* PCGs, twelve genes started with ATN, but nad1 initiates with TTG. Although most insect mitogenomes begin with ATN codons [73], the unusual initiation codon for the nad1 gene is also present in the mitogenomes of some other insects, such as *Liriomyza trifolii* (GTG) and *Agonita chinensis* (TTG) [32,41]. The cox1 gene begins with an ATN codon and is considered to be a characteristic of ancestral insects, although this still needs to be examined [71].

Complete stop codons (TAG and TAA) were found in 2 PCGs and 8 PCGs in *T. auricollis*, respectively. The remaining three genes appear to end with T or TA; two of these are adjacent to tRNAs, and one is located between nad4 and nad4l (Table 3). The incomplete stop codon may be converted into a proper TAA stop codon by RNA polyadenylation [76], which is common in animal genomes and can produce functional termination codons via polycistronic transcription cleavage and polyadenylation mechanisms [77]. The same stop codons are utilized in other PCGs, except nad5, of both *Trachys* species. The stop codon T located in nad5 of *T. troglodytiformis* is different from that of *T. auricollis*, which has a TAG stop codon. These differences between the two species might result from the 20 bp overlapping region between nad5 and trnF in *T. auricollis*; no such overlapping region exists in *T. troglodytiformis*.

Ka/Ks ratios are a powerful approach for testing the neutral evolution model [78]; these ratios have been used to diagnose the form of sequence evolution [79]. Evaluation of the Ka/Ks ratios for all PCGs of the two *Trachys* species revealed the atp8 and nad4l ratios to be larger than 1; atp8 has the highest evolutionary rate, and cox1 the lowest (Figure S2). The lowest A + T content in the cox1 gene might reflect its high conservation [72]. Indeed, cox1 shows the lowest Ka/Ks value (i.e., lowest evolutionary rate) in nearly all animals (e.g., crustaceans [80,81,82], insects [83,84,85,86,87], mollusks [88,89,90], birds [91,92], and mammals [93]), indicating that this gene should be generally under the highest purification/selection pressure and functional constraints [80]. cox1 is thus the best DNA barcode for species identification and phylogenetic resolution in animals [89]. atp8 is one of the genes with the highest Ka/Ks value (i.e., highest evolutionary rate) in many animals (e.g., crustaceans, [80,81,82], insects [84,85,86,87], mollusks [88,89,90], birds [91,92], and mammals [93]), indicating that atp8 should be generally under low purification/selection pressure and functional constraints, [80,88]. With a Ka/Ks value of atp8 and nad4l > 1, the two genes would be considered representative of positive selection with some advantageous mutations, though negative selection tended to be indicated for the other genes [94]. 

### 3.3. Codon Usage

RSCU values for the PCGs in the mitochondrial genomes of the two *Trachys* species were analyzed, with most differing from 1 (frequency at equilibrium). The five most frequently used codons in *T. auricollis* are UUA(L), CGA(R), AUA(M), AAA(K), and GCU(A), and the first two most frequently used codons are consistent with those of *T. troglodytiformis* (Figure S3). Previous research has indicated that NNA and NNU (N represents A, T, C, G) codons can be used to express the frequency of A + T bias in PCGs [39].

### 3.4. Transfer RNAs

The total length of the *T. auricollis* tRNAs is 1,444 bp, with each tRNA gene ranging in size from 60 bp (trnC) to 73 bp (trnW) (Table 3). The A + T content of the 22 tRNAs is 73.4%, ranging from 82.3% (trnD) to 63.7% (trnQ) (Table S1). Compared with *T. troglodytiformis*, *T. auricollis* has a larger total length of tRNAs (1,450 bp) and a higher A + T content of tRNAs (76%).

In the *T. auricollis* mitogenome, all 22 tRNA genes show typical cloverleaf structures, except for trnS1 (Figure S4). The same structures are found in *T. troglodytiformis*. For trnS1, the D-stem pairings in the dihydrouridine (DHU) arm are absent, as in many insect species (Figure S5). Although the trnS1 genes of both *Trachys* species are 67 bp in size and both UCUs are located in the anticodon loop (AC-loop), apparent differences can be observed in their structure; the structure of UCUs in the anticodon loop might be considered to be indicative of those of more ancient insect groups [95]. The D-loop of the *T. troglodytiformis* trnS1 gene contains six bases more than that of *T. auricollis*, which is composed of the nonclassical base-pair A-U. For all other beetles, the D-loop, T-loop, and T-stem are easily mutated, whereas the AC-loop maintains high conservation [72]. 

### 3.5. Ribosomal RNAs

The boundaries of rRNA genes are delineated based on the alignment of the two leaf-mining jewel beetles. The large ribosomal RNA (rrnl) gene of *T. auricollis* is 1294 bp in length, with an A + T content of 76.8%; the small rRNA (rrns) gene is 758 bp, with an A + T content of 75.2% (Table S1). The two rRNA genes mapped between the trnL1 and trnV and the trnV and A + T-rich regions (Figure 2 and Table 3). Compared with other jewel beetles, the two rRNA genes of *T. troglodytiformis* and *Chrysochroa fulgidissima* have similar locations [38].

### 3.6. A + T-Rich Region

The A + T-rich region (CR) of *T. auricollis* is located between rrnS and the trnI-trnG-trnM gene cluster (Figure 2 and Table 3). The CR of *T. auricollis* includes six 72 bp tandem repeats (14,795–14,865 bp), with approximately 10 bp of poly-A stretches, with 16 bp of poly-T stretches at the 3’ end of the CR. This region shows a 73.4% A + T composition and a length of 1,847 bp, which is slightly longer than that of *T. troglodytiformis* (1728 bp) (Figure S6), with an A + T content of approximately 78.9%. The A + T-rich region is the longest sequence in the mitogenomes of *T. auricollis* and *T. troglodytiformis*; however, the highest A + T content among all genes is not found in the A + T-rich region but rather in the rrnL gene (Table S1). This A + T-rich region length is well within the range of those of other beetles, displaying remarkable variability and spanning from 201 bp for *Dryops* sp. to 4,469 bp for *Coccinella septempunctata* (Coccinellidae) [68,96].

In contrast, *T. troglodytiformis* harbors different repeated sequence regions (15,861–15,902 bp) (Figure S7). Moreover, a conserved structural pattern was found in the two species. The size of the A + T-rich region might influence variation among beetle mitochondrial genomes [97], and the CR contains initiation sites for transcription and replication [98].

### 3.7. Intergenic Spacer and Overlapping Regions

Gene origin sites are almost immediately downstream of the 3’ end of the previous gene; however, the overlap may occasionally occur at some initiation sites. The total length of the 20 overlapping regions in the *T. auricollis* mitogenome is 147 bp, ranging from 1 bp to 30 bp (Table 3). The first three longest overlap regions in the *T. auricollis* mitogenome are located between trnH and nad4 (30 bp), trnL1 and rrnL (23 bp), and trnF and nad5 (20 bp). In addition to the largest CR, 135 bp of intergenic nucleotides [99] are present in 7 spacers, ranging from 2 bp (nad4l and trnT) to 39 bp (trnM and nad2), in *T. auricollis*.

In contrast, *T. troglodytiformis* harbors only 13 overlapping regions ranging from 1 bp to 8 bp and five intergenic regions ranging from 1 bp to 26 bp. Additionally, these mitogenomes differ in their longest overlapping and intergenic regions. Some of the overlapping regions in *T. auricollis* consist of the intergenic regions present in the mitogenome of *T. troglodytiformis*, such as the intergenic regions of nad2-trnW (5 bp) and nad4-nad4l (23 bp)*,* which are present at the overlapping regions in *T. troglodytiformis* at 1 bp and 7 bp, respectively.

### 3.8. Phylogenetic Analyses

**Figure 3:**
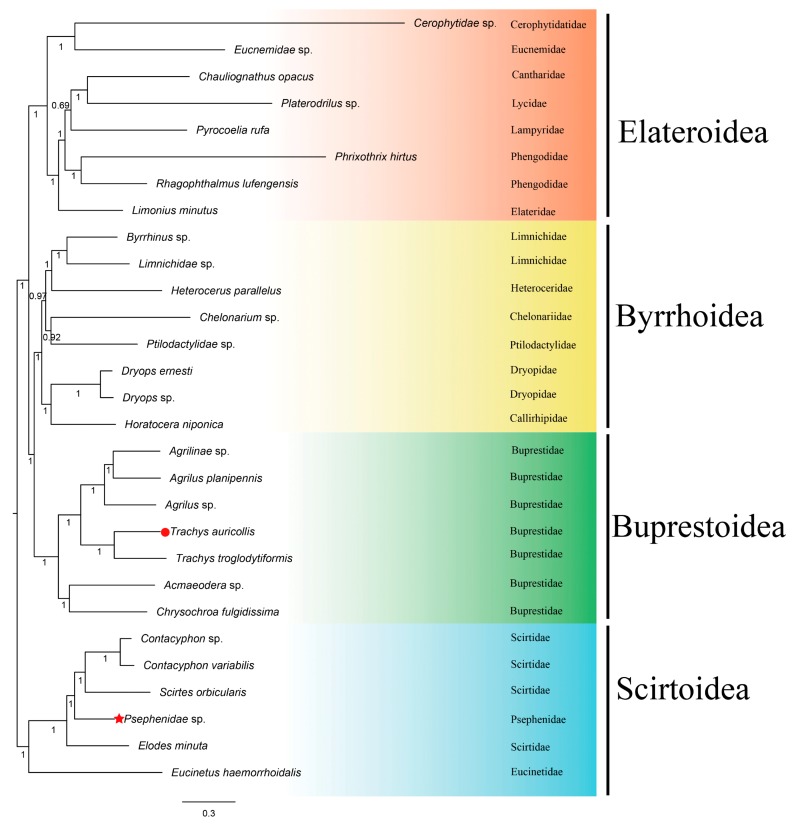
Maximum likelihood (ML) tree of evolutionary relationships between *T. auricollis* (solid red circle) and 27 other beetles based on all PCGs, all rRNAs, and all tRNAs. Red stars indicate inconsistent placement, as shown in Table 2. ML bootstrap values are shown at each node. The bar represents the number of substitutions per site.

**Figure 4:**
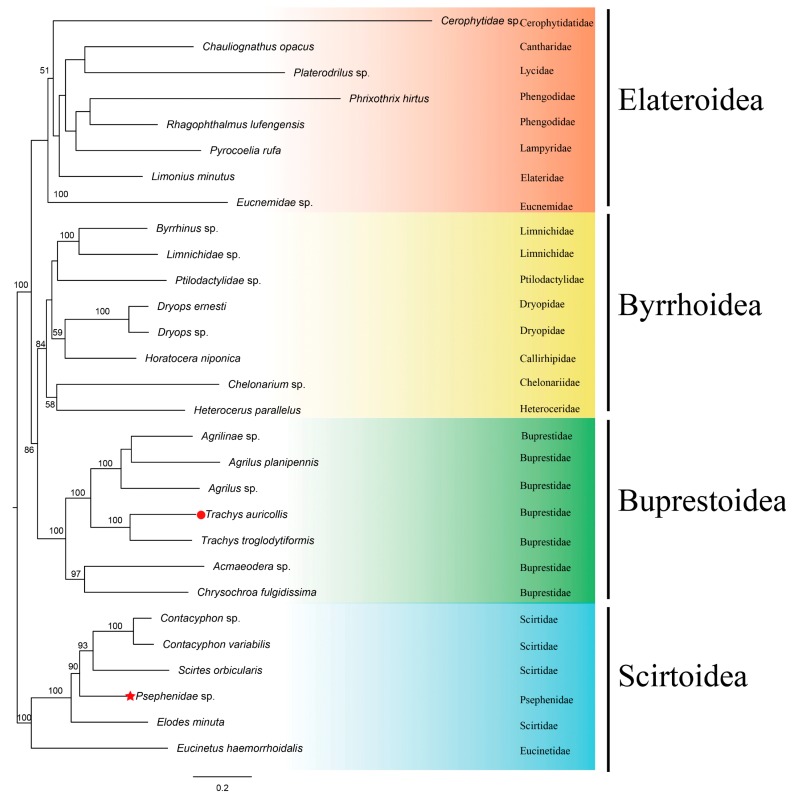
Bayesian (BI) tree of evolutionary relationships between *T. auricollis* (solid red circle) and 27 other beetles based on all PCGs, all rRNAs, and all tRNAs. Red stars indicate inconsistent placement, as shown in Table 2. Posterior probabilities are shown at each node. The bar represents the number of substitutions per site.

Phylogenetic relationships were established based on the concatenated amino acid sequences of all PCGs, all rRNAs, and all tRNAs for all available Elateriformia species using Scirtoidea as the outgroup and applying both ML and BI methods (Table 2 and Figure 3 and Figure 4). The log-likelihood (-LnL) value of the ML tree is 251,072, and the harmonic mean log-likelihood (-HMLi) value of the BI tree is 251,499.

In this study, the topologies of both trees were stable at the superfamily level. Both trees show that Buprestoidea (Buprestidae only, without Schizopodidae) and Byrrhoidea (excluding Psephenidae) are reciprocally monophyletic groups; Elateroidea clusters as a sister to a clade of Byrrhoidea and Buprestoidea, but Psephenidae (of Byrrhoidea) is located within the Scirtoidea group. Our phylogenetic results support that Buprestoidea is a monophylum that is close to Byrrhoidea [12,14,16,19,21,22,30]; Buprestoidea and Byrrhoidea cluster within a clade sister to Elateroidea [16,28,29,31], and the position of Psephenidae is undetermined [30]. 

There might be two possible ways to increase the accuracy of phylogenetic topological structure: one is to use more species, the other is to use more genes. The phylogenetic trees based on over 400 species all support that Buprestoidea and Byrrhoidea are very close, with Elateroidea located outside them (Figure 1 and Table 1) [28,29,30]. Our analysis with all 37 mitogenomic genes, including 13 PCGS, 22 tRNAs, and two rRNAs, agreed with this topology. That is, the topology based on either abundant species or abundant genes becomes consistent here. The inclusion of tRNAs might help to resolve the phylogeny of Coleoptera, just as in Diptera, Orthoptera, Neuropterida, and Lepidoptera [43,47,48,49]. 

However, due to the absence of complete mitogenomes for the Dascilloidea superfamily and several families in other superfamilies (such as Schizopodidae in Buprestoidea; Cneoglossidae, Elmidae, Eulichadidae, and Lutrochidae in Byrrhoidea; Artematopodidae, Brachypsectridae, Omalisidae, Omethidae, Podabrocephalidae, and Throscidae in Elateroidea; and Clambidae and Decliniidae in Scirtoidea), the placement of Buprestoidea in Elateriformia requires further verification. The Elateroidea appeared to have less support for internal nodes in the ML tree (Figure 3). Perhaps adding two representative species rather than one per family could help to stabilize the clustering pattern. However, we focus mainly on the relationships among different superfamilies in this study. Too many species in one superfamily might bias the topology. We hope that all the issues can be well solved when enough mitogenomes are accumulated for Elateriformia species in the future. 

## 4. Conclusions

The mitogenome of the leaf-mining jewel beetle *T. auricollis* is the largest among the reported jewel beetle mitogenomes. The data obtained in this study reveal a typical closed-circular and double-stranded DNA molecular structure. The AT skew, GC skew, base composition, Ka/Ks ratio, and RSCU of the genes were calculated, and secondary cloverleaf structures for tRNA genes were predicted. Initiation and stop codons, tandem repeated units, and intergenic spacer and overlapping regions were analyzed. Our whole-mitogenome phylogenetic results support that Buprestoidea is close to Byrrhoidea and that Buprestoidea and Byrrhoidea cluster within a clade sister to Elateroidea; nonetheless, the position of Psephenidae remains undetermined. The inclusion of tRNAs might help to resolve the phylogeny of Coleoptera. 

## Figures and Tables

**Table 1:** Molecular phylogenetic studies assessing the relationship of Buprestoidea with other Elateriformia superfamilies.

Taxonomic Level	Elateriformia Groups Used *	Genes Used	References
Coleoptera	4 superfamilies + Scirtoidea 30 families 704 species	rRNA: 18S, 28S mtDNA: rrnl, cox1(cox1-5, cox1-3’)	[28]
Coleoptera	4 superfamilies + Scirtoidea 33 families 59 species	rRNA: 18S, 28S nuclear: AK, AS, CAD, EF1a, PEPCK, WG	[16]
Coleoptera	4 superfamilies 7 families 34 morphospecies	mtDNA: 1–13 PCGs	[31]
Coleoptera	4 superfamilies + Scirtoidea 29 families 564 species	rRNA:18S, 28S mtDNA: rrnl, cox1 Transcriptomes: 4220 orthologs	[29]
Coleoptera	4 superfamilies + Scirtoidea 27 families 85 species	nuclear: 95 PCGs	[17]
Coleoptera	4 superfamilies 46 subfamilies 189 species	rRNA: 18S mtDNA: rrnl, cox1	[18]
Coleoptera	3 superfamilies 8 families 12 species	mtDNA: 12 or 13 PCGs	[25]
Elateriformia	4 superfamilies + Scirtoidea 28 families 112 species	rRNA: 18S, 28S mtDNA: rrnl, cox1	[27]
Elateriformia	4 superfamilies 17 families 27 species	mtDNA: 12 PCGs or cob-nad6	[26]
Elateriformia	4 superfamilies + Scirtoidea 31 families 488 species	rRNA: 18S, 28S mtDNA: rrnl, cox1	[30]
Elateriformia	3 superfamilies + Scirtoidea 19 species	mtDNA: all 13 PCGs	[23]
Elateriformia	3 superfamilies + Scirtoidea 18 species	mtDNA: all 13 PCGs	[24]
Elateriformia	3 superfamilies + Scirtoidea 18 families 31 species	mtDNA: all 13 PCGs, rrnl, rrnlS, 22 tRNA	this study

* Elateriformia are treated as the four-superfamily system, including Buprestoidea, Byrrhoidea, Elateroidea, and Dascilloidea [11,12,13].

**Table 2:** List of taxa used for the phylogenetic analysis in this study.

Superfamily	Family	Species*	GenBank NO.	Size (bp)	Total A + T%	AT% of all PCGs	References
Buprestoidea	Buprestidae	*Acmaeodera* sp.	FJ613420	16,217	68.4	66.2	[63]
Buprestoidea	Buprestidae	*Agrilus planipennis*	KT363854	15,942	71.9	70.1	[25]
Buprestoidea	Buprestidae	*Agrilus* sp.	JX412834	16,210	70.1	68.4	[64]
Buprestoidea	Buprestidae	*Chrysochroa fulgidissima*	NC012765	15,592	69.9	68.6	[38]
Buprestoidea	Buprestidae	*Trachys auricollis*	MH638268	16,429	71	69.3	This study
Buprestoidea	Buprestidae	*Trachys troglodytiformis*	KX087357	16,316	74.6	73.6	[65]
Buprestoidea	Buprestidae	Agrilinae sp.	MH789732	16,173	72.5	70.3	[31]
Byrrhoidea	Limnichidae	*Byrrhinus* sp.	JX412827	16,812	72.4	70.3	[64]
Byrrhoidea	Callirhipidae	*Horatocera niponica*	KX035160	16,107	75.5	73.4	[66]
Byrrhoidea	Dryopidae	*Dryops ernesti*	KX035147	15,672	73	71	[67]
Byrrhoidea	Dryopidae	*Dryops luridus*	KT876888	16,710	72.9	71.1	[68]
Byrrhoidea	Heteroceridae	*Heterocerus parallelus*	KX087297	15,845	74	72.5	[65]
Byrrhoidea	Limnichidae	Limnichidae sp.	JQ034416	14,388	74.6	73.5	[26]
Byrrhoidea	Psephenidae	Psephenidae sp.	KX035154	16,312	78.1	75.6	[66]
Byrrhoidea	Ptilodactylidae	Ptilodactylidae sp.	MH789727	15,991	74.8	72.1	[31]
Byrrhoidea	Chelonariidae	*Chelonarium* sp.	KX035150	15,095	75.6	72.9	[67]
Elateroidea	Cantharidae	*Chauliognathus opacus*	FJ613418	14,893	76.8	76.2	[63]
Elateroidea	Cerophytidae	Cerophytidae sp.	KX035161	15,741	80.4	79	[67]
Elateroidea	Elateridae	*Limonius minutus*	KX087306	16,727	76.7	74.8	[65]
Elateroidea	Lampyridae	*Pyrocoelia rufa*	AF452048	17,739	77.4	76.3	[69]
Elateroidea	Lycidae	*Platerodrilus* sp.	KU878647	16,394	76.9	76	[70]
Elateroidea	Phengodidae	*Phrixothrix hirtus*	KM923891	18,919	78	77.9	[34]
Elateroidea	Rhagophthalmidae	*Rhagophthalmus lufengensis*	NC010969	15,982	79.6	78.1	[35]
Elateroidea	Eucnemidae	Eucnemidae sp.	MH923241	16,170	78.3	76.2	[31]
Scirtoidea	Scirtidde	*Cyphon* sp.	NC011320	15,919	75.2	72.8	[71]
Scirtoidea	Scirtidde	*Contacyphon variabilis*	KT876886	15,901	75.9	71.1	[68]
Scirtoidea	Scirtidde	*Elodes minuta*	KX087288	17,043	76.8	72.8	[65]
Scirtoidea	Eucinetidae	*Eucinetus haemorrhoidalis*	NC036278	17,954	81	78.4	[67]
Scirtoidea	Scirtidae	*Scirtes orbicularis*	KX087343	13,944	76.5	75.4	[65]

* The mitogenome sequence of a Scirtidae sp. (KT696212) was not included because it was close to Staphylinoidea species and far from other Scirtoidea species when blast-searched in NCBI.

**Table 3:** Summary of the mitogenome of *T. auricollis.*

Feature	Strand	Position	Length (bp)	Initiation Codon	Stop Codon	Anticodon	IGN
trnI	N	1–67	67			GTA	−3
trnQ	J	65–133	69			TTG	
trnM	N	134–202	69			CAT	39
nad2	N	242–1222	981	ATG	TAA		5
trnW	N	1228–1300	73			TCA	−8
trnC	J	1293–1352	60			GCA	
trnY	J	1353–1417	65			GTA	−8
cox1	N	1410–2954	1,545	ATT	TAA		−5
trnL2	N	2950–3014	65			TAA	
cox2	N	3015–3696	682	ATA	T(AA)		−3
trnK	N	3694–3764	71			CTT	−2
trnD	N	3763–3824	62			GTC	
atp8	N	3825–3983	159	ATT	TAA		−7
atp6	N	3977–4651	675	ATG	TAA		−1
cox3	N	4651–5437	787	ATG	T(AA)		
trnG	N	5438–5499	62			TCC	
nad3	N	5500–5883	354	ATA	TAG		−2
trnA	N	5852–5914	63			TGC	−1
trnR	N	5914–5980	67			TCG	−1
trnN	N	5980–6044	65			GTT	
trnS1	N	6045–6111	67			TCT	
trnE	N	6112–6173	62			TTC	−1
trnF	J	6173–6235	63			GAA	−20
nad5	J	6216–7934	1,719	ATT	TAG		18
trnH	J	7953–8015	63			GTG	−30
nad4	J	7986–9321	1,336	ATG	T(AA)		23
nad4l	J	9345–9632	288	ATG	TAA		2
trnT	N	9635–9697	63			TGT	−1
trnP	J	9697–9762	66			TGG	−8
nad6	N	9755–10252	498	ATT	TAA		−1
cob	N	10252–11397	1,146	ATG	TAA		−2
trnS2	N	11396–11462	67			TGA	23
nad1	J	11486–12412	927	ATT	TAA		25
trnL1	J	12438–12502	65			TAG	−23
rrnL	J	12480–13773	1,294				−19
trnV	J	13755–13824	70			TAC	
rrnS	J	13825–14582	758				1847
CR	-	14582–16429	1,846				
Genome Size		16429					0

J and N refer to the major and minor strands, respectively. Position numbers refer to positions on the majority strand. CR = the control region is also named the A + T-rich region. IGN = intergenic nucleotides.

## Supplementary Materials

The following are available online at https://www.mdpi.com/2073-4425/10/12/992/s1, Figure S1: The base composition of protein-coding genes and two rRNAs in the mitochondrial genomes of *T. auricollis* and *T. troglodytiformis*, Figure S2. Ka/Ks ratios of 13 protein-coding genes. Ka is the nonsynonymous substitution rate, and Ks is the synonymous substitution rate, Figure S3. Relative synonymous codon usage (RSCU) for protein-coding genes of *T. auricollis* and *T. troglodytiformis* mitochondrial genomes. Codon families are provided on the x-axis, Figure S4. Predicted secondary structures of the 22 typical tRNA genes of the *T. auricollis* mitochondrial genome, Figure S5. Predicted secondary cloverleaf structure for the trnS1 genes of *T. auricollis* and *T. troglodytiformis,* Figure S6. Alignment of the conserved structural elements of the control regions (CRs) of *T. auricollis* and *T. troglodytiformis*, Figure S7. Partial A + T-rich regions of *T. auricollis* and *T. troglodytiformis*. The underlined sequences are perfectly repeated sequences in the A + T-rich region. The position refers to the length of the first repeated sequence, Table S1: Length, A + T content (%), AT skew, and GC skew for *T. auricollis* and *T. troglodytiformis.*
