# Supplementary material for: Complete Mitogenome of a Leaf-Mining Buprestid Beetle, Trachys auricollis, and Its Phylogenetic Implications
Source: Genes (Basel). 2019 Dec 1;10(12):992. doi: 10.3390/genes10120992 (PMC6947639; doi:10.3390/genes10120992)
Supplement: Supplementary file 1 [file genes-10-00992-s001.pdf]

Table S1. Length, A + T content (%), AT skew, and GC skew for *T. auricollis* and *T. troglodytiformis.*

Region	*Trachys Auricollis*				*Trachys Troglodytiformis*			
	Length (bp)	A + T (%)	AT Skew	GC Skew	Length (bp)	A + T (%)	AT Skew	GC Skew
genome	16429	71.1	0.10	−0.20	16316	74.6	0.10	−0.19
PCGs	11097	69.4	−0.14	−0.02	11134	73.0	−0.14	−0.02
rrnl	1294	76.8	−0.15	0.31	1260	79.2	−0.13	0.32
rrns	758	75.2	−0.07	0.27	757	77.3	−0.10	0.27
CR	1847	73.4	0.04	−0.22	1728	78.9	0.16	−0.26
tRNA	1444	73.4	0.02	0.11	1450	76.0	0.01	0.12

AT skew = (A − T)/(A + T), GC skew = (G − C)/(G + C); CR = control region, also called the A + T-rich region.

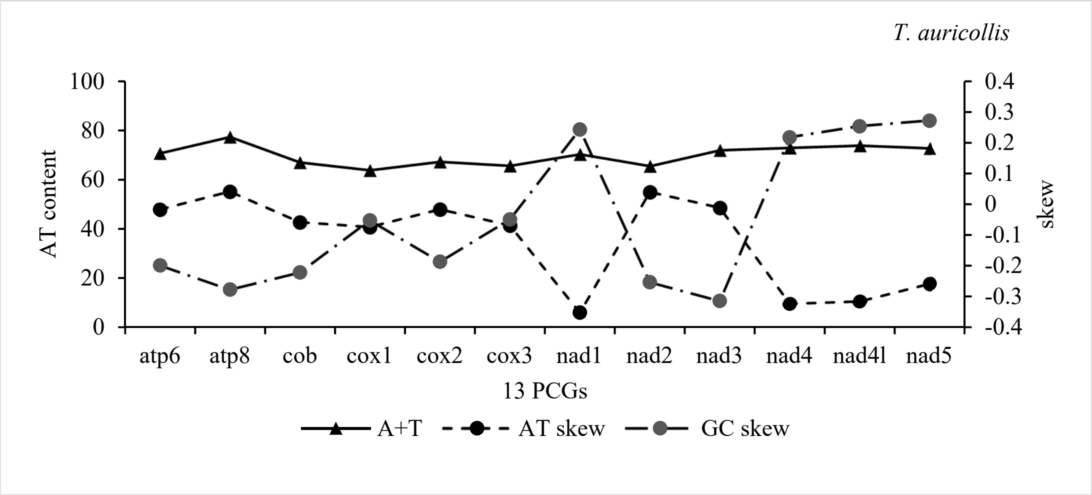

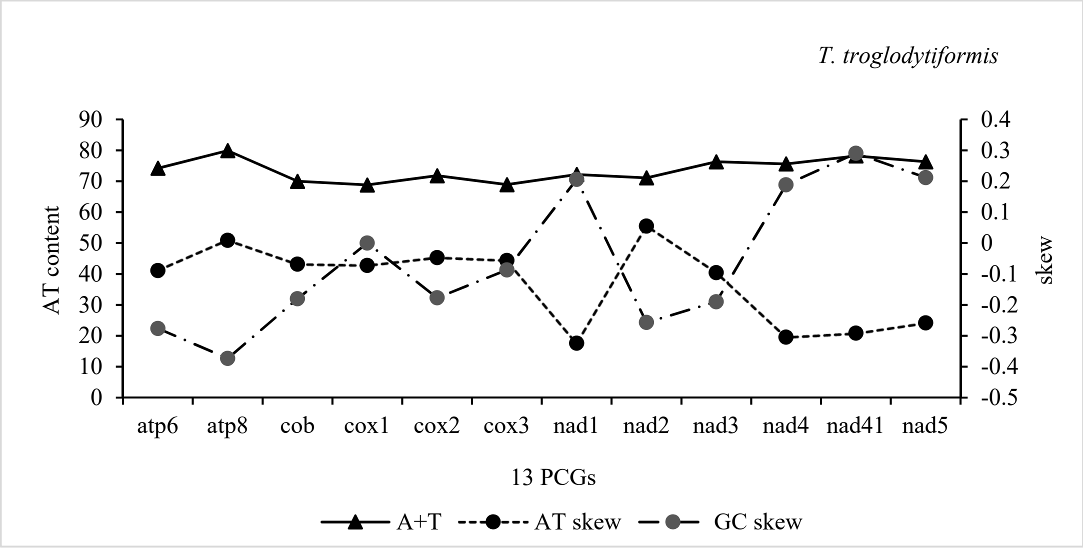

Figure S1. The base composition of protein-coding genes and two rRNAs in the mitochondrial genomes of *T. auricollis* and *T. troglodytiformis*.

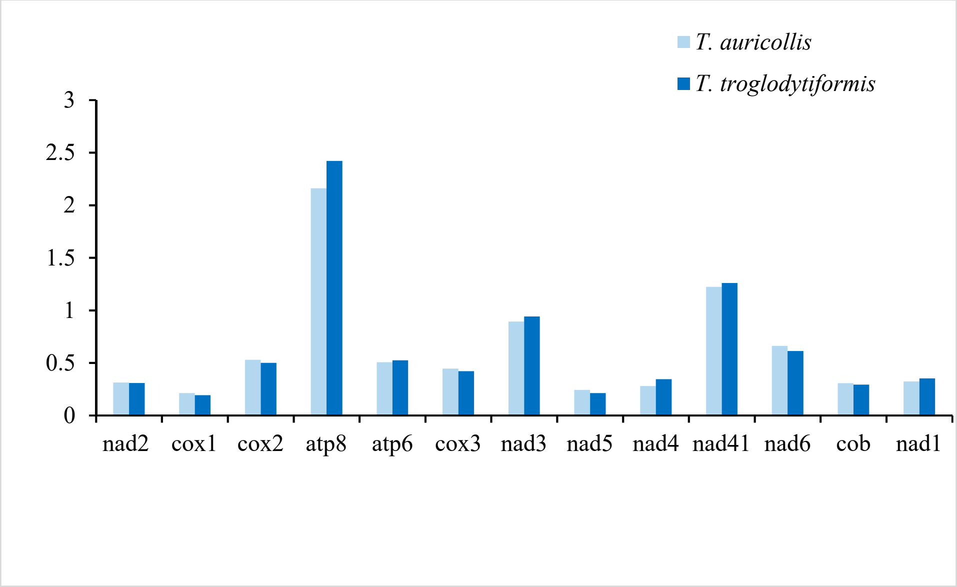

Figure S2. Ka/Ks ratios of 13 protein-coding genes. Ka is the nonsynonymous substitution rate, and Ks is the synonymous substitution rate.

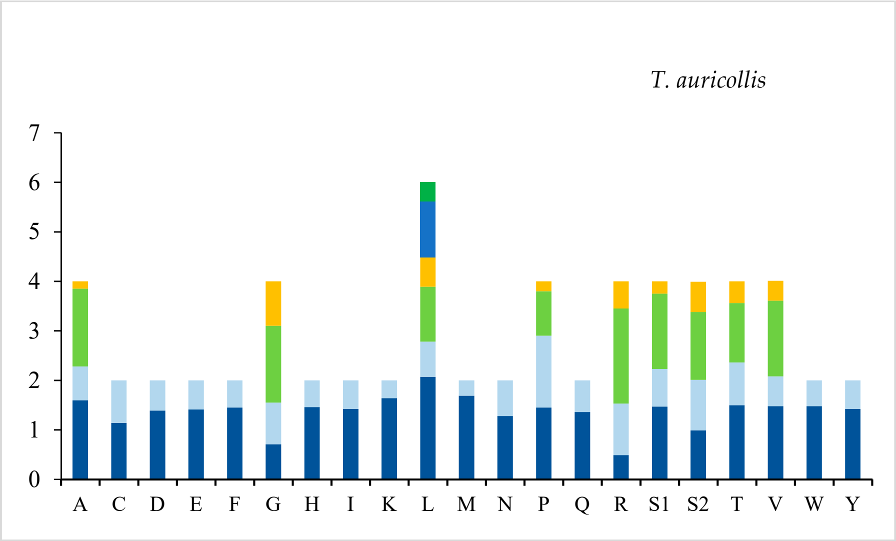

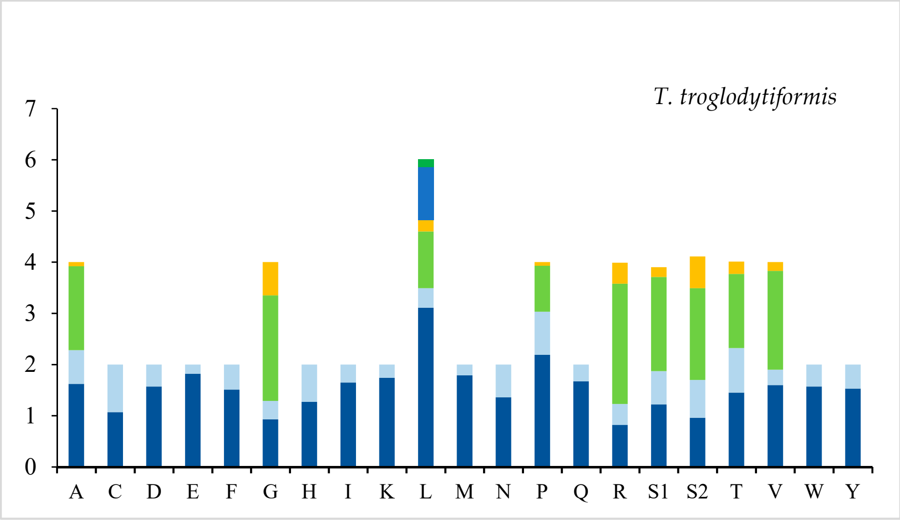

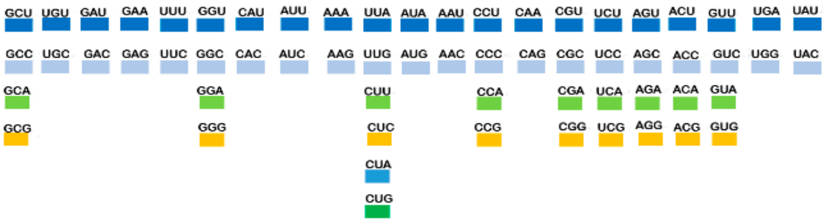

Figure s3. Relative synonymous codon usage (RSCU) for protein-coding genes of *T. auricollis* and *T. troglodytiformis* mitochondrial genomes. Codon families are provided on the x-axis.

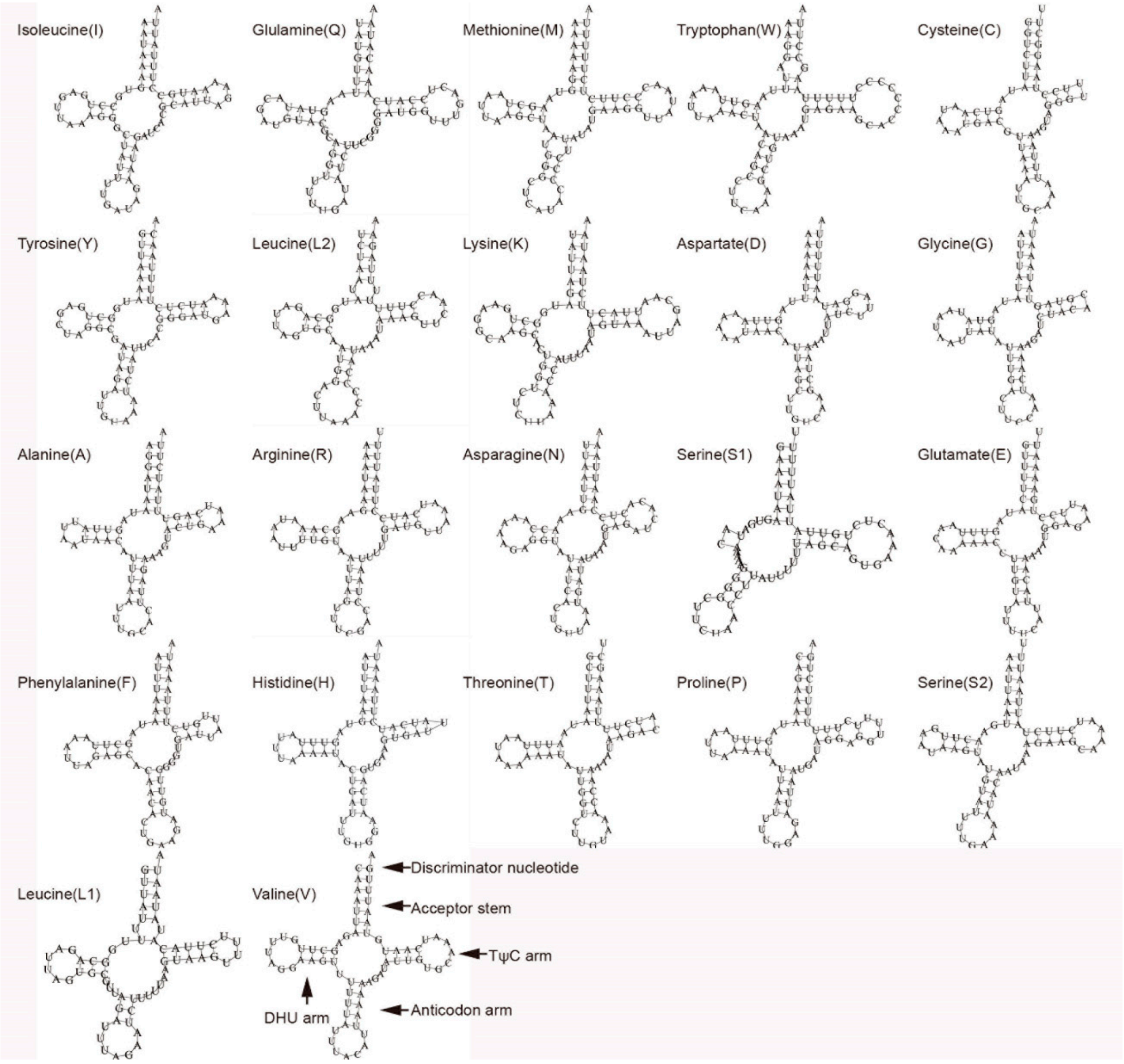

Figure S4. Predicted secondary structures of the 22 typical tRNA genes of the *T. auricollis* mitochondrial genome.

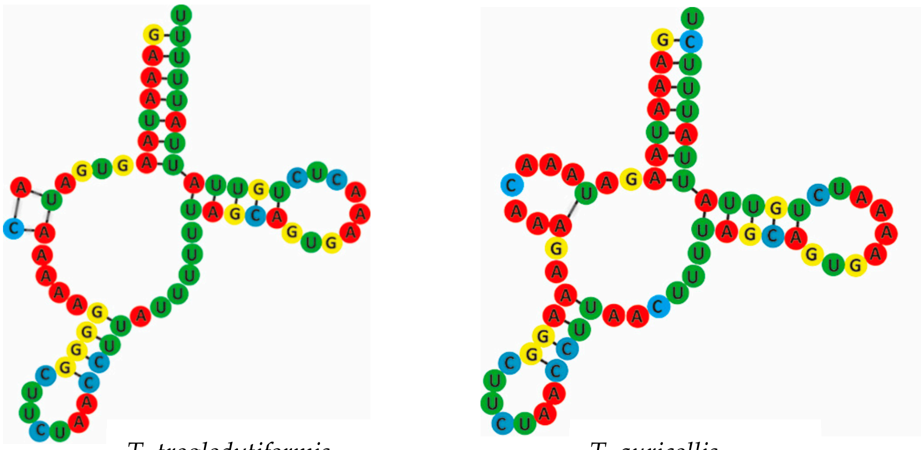

T. troglodytiformis *T. auricollis*

Figure S5. Predicted secondary cloverleaf structure for the trnS1 genes of *T. auricollis* and *T. troglodytiformis*. Nucleotide sequences from 5′ to 3′ are indicated for trnS1, Watson-Crick base-pairing, and G-U base-pairing. The arms of tRNAs (clockwise from top) are the amino acid acceptor (AA) arm, TΨC (T) arm, the anticodon (AC) arm, and the dihydrouridine (DHU) arm.

```
T. auricollis         - G T T A A G T T A G A C T T A T T A A C A A - - - T T T G
T. troglodytiformis   A A A C A A G T T T A A A A T A A C A A C A A A G A T T T A

T. auricollis         T C T A T T T T T G T T A C T A A A A T T T A T T A A C G A
T. troglodytiformis   A A T A T T T A T A T T A C T A A A T T T T A T T A A C G A

T. auricollis         T A A A G A T A T T G T G T T A A T G A C T A A T A A A C A
T. troglodytiformis   T A A T T A A A A G G T T A A A C C A A A T A A T A A A - G

T. auricollis         T A G C C C T G A C A G C T A A A G T A A G A T T T T C G A
T. troglodytiformis   T G A C C T C C C T G G T A A A A A C A A - - - C C T A G A

T. auricollis         T G A C A G C T A A A G T A A G A T T T T C G A C T C T A G
T. troglodytiformis   - A A C A A C C A G G G A A - - - - - C C T G A C - C T C C

T. auricollis         C T A A A G T A A G A T T T T C G A C T C T A G C T A G C T
T. troglodytiformis   C C C T G G T A A A A A A - - - - - - - - C A A C C T A G A A

T. auricollis         T A A G A T T T T C G A C T C T A G C T A G C T A A T C C C
T. troglodytiformis   T A A - - - C C T A G A A A C - A A C T A G G G A A - - C C

T. auricollis         T T T C G A C T C T A G C T A G C T A A T C C C T G A T A G
T. troglodytiformis   G T C A G A A T G A A C C T T A A A A A C C C C T T - T A A

T. auricollis         C T C T A G C T A G C T A A T C C C T A A T A G A T T A A C
T. troglodytiformis   C - - - - - - - - - - - - A T C C T C T T C A A A A C C A A

T. auricollis         C C C A A A C T C G C A G G T T T C A C C C C C C A A A A A
T. troglodytiformis   T T A A A A A T T - - - - - T T T T T T T T G C A A A A A

T. auricollis         A A T T T T T G A T T T T T A A A A A T A A C G A T A A T C
T. troglodytiformis   A A C C A A A T A A A A A A A A A A A A A A C A A - A A T T

T. auricollis         T T T T T T C C T C C A A A G A A T T T A T T C G G A A A A
T. troglodytiformis   T T T T T T T T T A T A A A A A A T - - A A T T A A A A A A

T. auricollis         A A A G C C C T A C C T T A C T A G G G C T T T C T C G T T
T. troglodytiformis   A A T T A C C A A A A T A A C T G A A G A A A A A C C G T T

T. auricollis         T A G T C G C A A T T C T A T A C A A A A A A G A A G A T T
T. troglodytiformis   T A A C G A C T T T C G T T C T C A C A C T A A A A A A T A

T. auricollis         G T C T G A T T T A A G T T C A A A A A A A A T T T T T T A
T. troglodytiformis   T C T T G T T T C A A G T T T G G G A A A T A T T T T T T T

T. auricollis         T T A A T A A T T T G T T T A C T A A A T T T A G T T A A C
T. troglodytiformis   A T A - T C T T T T A T T T A C T A A A A T T A G T T A A C

T. auricollis         A T A C A T A T T T T T T T T T T T T T T T T - - - - - C T
T. troglodytiformis   A G A C A T A T T T T T T T T T T T T T T T T T T T T T C T

T. auricollis         A A T T A T A A A C A A A T T A G A A G G G A T A A A T T T
T. troglodytiformis   A A T T A T A T A T T A A T T A A A A G A A A T A A A T T T

T. auricollis         A T A T A T T A A A A T G T T T A T A T A T A T A T T T A A
T. troglodytiformis   A T G A A T T A A A T A T C T T A T A T A T A T A T A T A A

T. auricollis         A C G A T A C G G A T T T A T A A A G T A T T C T A A T A T
T. troglodytiformis   A C G A T A A G G A T C C A C T A A T A T T A A G A A T A T

T. auricollis         A A T A A A C G A T G T T A C A T A G A G T T A A A T A T A
T. troglodytiformis   A A T A A A A T A T G T T A A A T A A G G T A A A A T A T A

T. auricollis         C C C C C C C T A A A A A T T A G C T T C T C C G T T T T C
T. troglodytiformis   C C C A T T T T T A A A A C T A C T A A A A G T A T T T T T

T. auricollis         T G C T - C G T T T A T A A A G G G A C T T T T T A T C A G
T. troglodytiformis   C C T T G C A G A T A G A A A A C G C C T T T T A A A C A G

T. auricollis         T A G C T A T A T A T T C A T T T T A A A A T T G G T T A G
T. troglodytiformis   T T A A T A - A C A A A A A A T A T A A C A T - A A A T A T

T. auricollis         A
T. troglodytiformis   -
```

Figure S6. Alignment of the conserved structural elements of the control regions (CRs) of *T. auricollis* and *T. troglodytiformis*. Sequence identity among species is indicated by yellow color.

T. auricollis

AAAAACCTAAAATGTAAATAACCAACAATAAATTAGCTTTAATCTTACGTAGCCCTGACAGCTAA
AGTAAGATTTTCGACTCTAGCTAGCTAATCCCTGACAGATTAGCTTTAATCATACGTAGCCCTGA
CAGCTAAAGTAAGATTTTCGACTCTAGCTAGCTAATCCCTGACAGATTAGCTTTAATCATACGTA
GCCCTGACAGCTAAAGTAAGATTTTCGACTCTAGCTAGCTAATCCCTGACAGATTAGCTTTAATC
TTACGTAGCCCTGACAGCTAAAGTAAGATTTTCGACTCTAGCTAGCTAATCCCTGACAGATTAGC
TTTAATCATACGTAGCCCTGACAGCTAAAGTAAGATTTTCGACTCTAGCTAGCTAATCCCTGATA
GATTAGCTTTAATCTTACGTAGCCCTGACAACTAAAGTAAGATTTTCGACTCTAGCTAGCTAATC
CCTAATAGATTAACTATTTAAACGAAAAATAATAGGGAGAATAAGATTCCCACTTAAAATAACC
CAAACTCGCAGGTTTCACCCCCCAAAAATCTCAAAAAAT Position(14,795-14,865)

T. troglodytiformis

ATACAGGAATTATTAAACAACCAGGGAACCTGACCTCCCTGGTAAAAACAACCTAGAAACAAC
CAGGGAACCTGACCTCCCTGGTAAAAACAACCTAGAAACAACCAGGGAACCTGACCTCCCTGG
TAAAAACAACCTAGAAACAACCAGGGAACCTGACCTCCCTGGTAAAAACAACCTAGAAACAAC
CAGGAAACCTGACCTCCCTGGTAAAAATAACCTAGAAACAACTAGGGAACCTAACTATTAAATT
AAAAATATTAAAAATAAAATGTCAGAATGAACCTTAAAAACCCCTTTAAATCAACCAGAAATAA
AAGAAATCATAAACCAAAAAATCTGACATCCTCTTCAAAACCAAAATTCAGTAAACACAAAAGA
CCTAAAAAAAAAATTTTTCCAAAATTTTTAAAAATTTTTTTTTTTGCAAAAAAAAATTTTTTTTTTTT
AAAAATTCATTAAAAAACCAAATAAAAAAAAAAAAAAACAAAATT Position(15,861-15,902)

Figure S7. Partial A + T-rich regions of *T. auricollis* and *T. troglodytiformis*. The underlined sequences are perfectly repeated sequences in the A + T-rich region. The position refers to the length of the first repeated sequence.
